# The differences of the economic losses due to presenteeism and treatment costs between high‐stress workers and non‐high‐stress workers using the stress check survey in Japan

**DOI:** 10.1002/1348-9585.12346

**Published:** 2022-07-07

**Authors:** Tomohisa Nagata, Ryotaro Ito, Masako Nagata, Kiminori Odagami, Shigeyuki Kajiki, Kenji Fujimoto, Shinya Matsuda, Koji Mori

**Affiliations:** ^1^ Department of Occupational Health Practice and Management, Institute of Industrial Ecological Sciences University of Occupational and Environmental Health Kitakyushu Japan; ^2^ Department of Occupational Medicine School of Medicine University of Occupational and Environmental Health Kitakyushu Japan; ^3^ Occupational Health Data Science Center University of Occupational and Environmental Health Kitakyushu Japan; ^4^ Department of Preventive Medicine and Community Health, School of Medicine University of Occupational and Environmental Health Kitakyushu Japan

**Keywords:** dental cost, high‐stress, medical cost, presenteeism, stress check

## Abstract

**Objectives:**

This study sought to examine differences in the economic losses due to presenteeism and costs of medical and dental treatment between high‐stress workers and non‐high‐stress workers using the stress check survey.

**Methods:**

We conducted a cross‐sectional study from April 1, 2018 to March 31, 2019 in a pharmaceutical company. High‐stress workers were classified with the Brief Job Stress Questionnaire using two methods: the sum method and the score converted method. The incidence of presenteeism and its costs were determined using a questionnaire. The costs of medical and dental treatment were calculated according to claims. We compared the costs between high‐stress and non‐high‐stress workers using Wilcoxon's rank‐sum test.

**Results:**

Of 3910 workers, 6.3% were classified as high‐stress using the sum method and 6.6% were classified as high‐stress using the score converted method. The costs associated with presenteeism and medical treatment among high‐stress workers were higher than the costs among non‐high‐stress workers, whereas the costs associated with dental treatment were not.

**Conclusions:**

To motivate employers to improve stressful work environments, it is recommended that presenteeism measurement items be added to the stress check survey, and that the methods used in this study be used to calculate the loss associated with high‐stress workers in Japanese companies. However, we must be careful in interpreting absolute presenteeism loss amounts because they are poorly reliable and valid.

## 1 INTRODUCTION

Occupational stress is a major risk factor not only for mental disorders but also for a variety of other health problems.^1^, ^2^, ^3^, ^4^ In recent years, international organizations and various national governments have proposed and developed programs aiming to prevent stress‐related diseases in workplaces.^5^, ^6^, ^7^, ^8^, ^9^ In Japan, the “Guidelines for Promoting Mental Health Care in Enterprises” were issued based on a provision of the Industrial Safety and Health Act in 2000,^10^ and the Occupational Safety and Health Act was amended to introduce a Stress Check Program, which obliges employers to conduct stress checks in workplaces employing 50 or more workers in 2015.^11^ Employers are expected to understand that the main purpose of such policy programs is to reduce occupational stress and prevent adverse health effects of workers.

The stress check program introduced in Japan consists of the following main elements: (a) providing an opportunity for an annual stress survey for all employees, and access to its results; (b) providing an interview with a physician for high‐stress workers at their request; and (c) improving the psychosocial work environment based on group analysis of data collected in the stress surveys.^12^ The Ministry of Health, Labour and Welfare (MHLW) published the Stress Check Manual regarding specific implementation methods, and recommends the use of the Brief Job Stress Questionnaire (BJSQ) for stress surveys and participatory workshops to improve the work environment.^13^

Although the evidence for the effectiveness of annual stress surveys as a measure of workers' mental health is weak,^14^ the effectiveness of participatory workplace improvement workshops at the organizational level has been confirmed.^15^, ^16^, ^17^ Nevertheless, a study conducted by the MHLW in 2020 reported that, when limited to workplaces employing 50 or more workers, stress check surveys were conducted in 91.5% of workplaces and group analysis was conducted in 81.1% of workplaces, whereas the implementation rate of participatory workshops to improve the work environment was only 7.6%.^18^ Thus, the stress check program is not currently effectively used for improving occupational stress.

One potential reason for the lack of progress in the implementation of participatory workshops is that the efforts require time for workers to participate and there is a cost associated with implementing improvements. If the impact of the presence of high‐stress workers in a stress check survey on business performance could be expressed in terms of monetary value, this may improve employers' understanding of the value of the efforts of improving occupational stress using participatory workshops. In Japan, where the population is aging, health and productivity management initiatives led by the government are being developed to improve labor productivity through health promotion,^19^ and the efforts of improving occupational stress are consistent with the health and productivity management initiatives. One of the prerequisites for the widespread use of participatory workshops and other measures to improve workplaces is that management understands the value and effectiveness of such measures for improving occupational stress.

Previous studies have reported that losses caused by presenteeism are much greater than the losses and medical costs associated with sickness absences caused by health problems among workers, and that psychological symptoms are the main symptoms causing presenteeism.^20^ Occupational stress causes productivity losses via presenteeism,^21^, ^22^ and both job stressors and social support directly and indirectly affected presenteeism.^23^, ^24^ In addition, it was reported that high‐stress workers judged by the stress check survey had higher productivity loss than non‐high stress workers,^25^ and approximately 75% of high‐stress workers exhibited mild work functioning impairment.^26^ However, there are only a few papers that examined the economic impact of the existence of high‐stress workers identified using a stress check survey from employers' aspect, including the associated medical costs and losses in labor productivity.

In the present study, we calculated the economic losses due to presenteeism and the costs of medical and dental treatment separately for high‐stress workers and non‐high‐stress workers and conducted comparisons between the two groups.

## 2 MATERIALS AND METHODS

**FIGURE 1:**
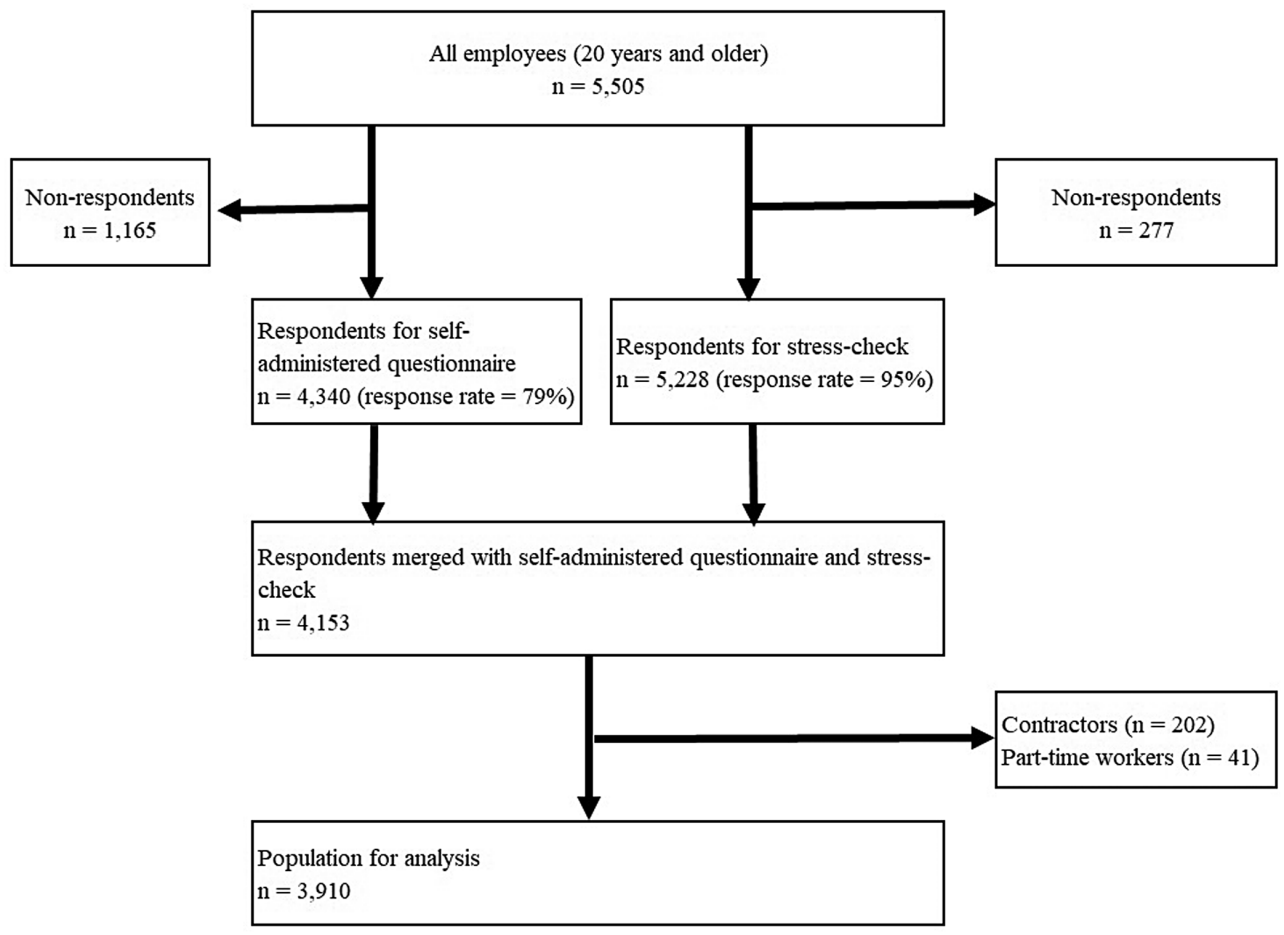
Flow diagram of this study

We conducted a cross‐sectional study of all employees (*n* = 5505) aged ≥20 years old in a pharmaceutical company and examined health insurance data in the 2018 fiscal year. We used three kinds of data: self‐administered questionnaire, stress check survey, and medical/dental claims. We conducted a self‐administered questionnaire to evaluate presenteeism in July and August, 2018, with 4340 respondents (response rate = 79%). The company conducted a stress check survey in July, 2018, with 5228 respondents (response rate = 95%). We merged the questionnaire data, survey data, and medical/dental claims data, and included 3910 workers in the final analysis after excluding contractors and part‐time workers (Figure 1).

This study was approved by the ethics committee of the University of Occupational and Environmental Health, Japan (H26‐026).

To identify high‐stress workers, the BJSQ for stress survey is recommended by the manual issued by the Japanese government.^13^ The manual recommends two criteria for classifying individuals as high‐stress: the sum method (simply summing the scores for each scales) and the score converted method (using converted scores according to the conversion table for each scales). The criteria are set so that the number of high‐stress workers is approximately 10% of the total.^13^ Employees identified as high‐stress exhibited significantly elevated risks for long‐term sickness absence during 1‐year follow‐up.^27^ Compared with the sum method, 1.5% more high‐stress workers were observed using the converted method.^28^

We evaluated presenteeism using a web‐based, self‐administered questionnaire. We asked participants whether they had experienced health issues at work over the preceding month. If the answer was yes, we asked how many days in a month they experienced symptoms, and whether the symptoms affected the quality and quantity of their work in comparison with productivity during periods without symptoms. The quality and quantity were scored on a scale from 0 to 10.^29^, ^30^ We calculated the costs due to presenteeism using the following formula:

$$\begin{array}{c} \text{Costs}\;\mathrm{due}\;\text{to presenteeism}=\text{Japanese}\;\mathrm{Yen}\;\left(\mathrm{JPY}\right)\;3300\\ \times 8\;\left(\text{working hours}\;\mathrm{per}\;\mathrm{day}\right)\times \left(1-\text{work}-\text{quantity}\;\left(0–10\right)\right)\\ \times \text{work}-\text{quality}\;\left(0–10\right)/100\\ \times \left(\text{days with symptoms in}\;a\;\text{month}\right)\\ \times 12\;\left(\text{to convert to the cost}\;\mathrm{per}\;\text{year}\right). \end{array}$$

The mean payroll per person per hour was set at JPY 3300 based on a previous study.^20^

We received medical and dental claims for all participants from the health insurance society, which covered the period between April 1, 2018 and March 31, 2019. Medical claims included medical treatment and drug administration for outpatients and inpatients. We calculated the costs of medical treatment and dental treatment directly from the claims.

We retrieved the following data from the self‐administered questionnaire for inclusion as control variables: age, sex, education, marital status and occupation. Age was treated as a continuous variable. Education was classified into four categories, as follows: junior high or high school; vocational school, junior college or technical school; university; and graduate school. Marital status was classified into four categories, as follows: married; unmarried (living alone); unmarried (living with family); and bereaved/divorced. Occupation was classified into six categories: clerical administrative support; sales; research; development; product line; and other.

### 2.1 Statistical analysis

Sex, education, marital status, and occupation were compared for high‐stress workers and non‐high‐stress workers using a chi‐square test. Similarly, age was compared with a *t*‐test.

We created two graphs of costs related to presenteeism, medical treatment, and dental treatment per person per year for high‐stress and non‐high‐stress workers using the two methods. We calculated the median and interquartile range, and performed Wilcoxon's rank‐sum tests for these costs to compare high‐stress with non‐high‐stress workers. All tests were two‐tailed, with differences reported as significant if *p* < .05. All analyses were performed in Stata version 16 (StataCorp).

## 3 RESULTS

We included 3910 of the 5505 employees of a pharmaceutical company in the analysis. A detailed flow diagram is shown in Figure 1. Demographic characteristics of the study population are shown in Table 1. The sample included 869 women (22%). The sum method identified 247 high‐stress workers (6.3%), and the score converted method identified 258 high‐stress workers (6.6%).

**TABLE 1:** Demographic characteristics of the study population

	Total	Sum method			Score converted method		
		Non‐high‐stress	High‐stress	*p* value	Non‐high‐stress	High‐stress	*p* value
Total	3910	3663	247		3652	258	
Sex (*N*, %)				.056^a^			.015^a^
Women	869 (22.2%)	802 (21.9%)	67 (27.1%)		796 (21.8%)	73 (28.3%)	
Age (mean, standard deviation)	44.7 (9.8)	44.8 (9.8)	43.2 (9.5)	.016^b^	44.8 (9.8)	43.0 (9.4)	.005^b^
Education (*N*, %)				.13^a^			.044^a^
Junior high or high school	374 (9.6%)	348 (9.5%)	26 (10.5%)		346 (9.5%)	28 (10.9%)	
Vocational school, junior college or technical school	185 (4.7%)	166 (4.5%)	19 (7.7%)		164 (4.5%)	21 (8.1%)	
University	2195 (56.2%)	2063 (56.4%)	132 (53.4%)		2061 (56.5%)	134 (51.9%)	
Graduate school	1149 (29.4%)	1079 (29.5%)	70 (28.3%)		1074 (29.5%)	75 (29.1%)	
Marital status (*N*, %)				<.001^a^			.001^a^
Married	2940 (75.8%)	2780 (76.4%)	160 (65.8%)		2772 (76.4%)	168 (65.9%)	
Unmarried (living alone)	618 (15.9%)	559 (15.4%)	59 (24.3%)		557 (15.4%)	61 (23.9%)	
Unmarried (living with family)	210 (5.4%)	197 (5.4%)	13 (5.3%)		194 (5.4%)	16 (6.3%)	
Bereaved/divorced	113 (2.9%)	102 (2.8%)	11 (4.5%)		103 (2.8%)	10 (3.9%)	
Occupation (*N*, %)				.002^a^			.015^a^
Clerical administrative support	480 (12.3%)	443 (12.1%)	37 (15.0%)		445 (12.2%)	35 (13.6%)	
Sales	1890 (48.3%)	1790 (48.9%)	100 (40.5%)		1782 (48.8%)	108 (41.9%)	
Research	517 (13.2%)	495 (13.5%)	22 (8.9%)		492 (13.5%)	25 (9.7%)	
Development	329 (8.4%)	297 (8.1%)	32 (13.0%)		299 (8.2%)	30 (11.6%)	
Product line	223 (5.7%)	206 (5.6%)	17 (6.9%)		206 (5.6%)	17 (6.6%)	
Others	471 (12.0%)	432 (11.8%)	39 (15.8%)		428 (11.7%)	43 (16.7%)	

^a^ Chi‐squre test.

^b^ *t* ‐test.

**FIGURE 2:**
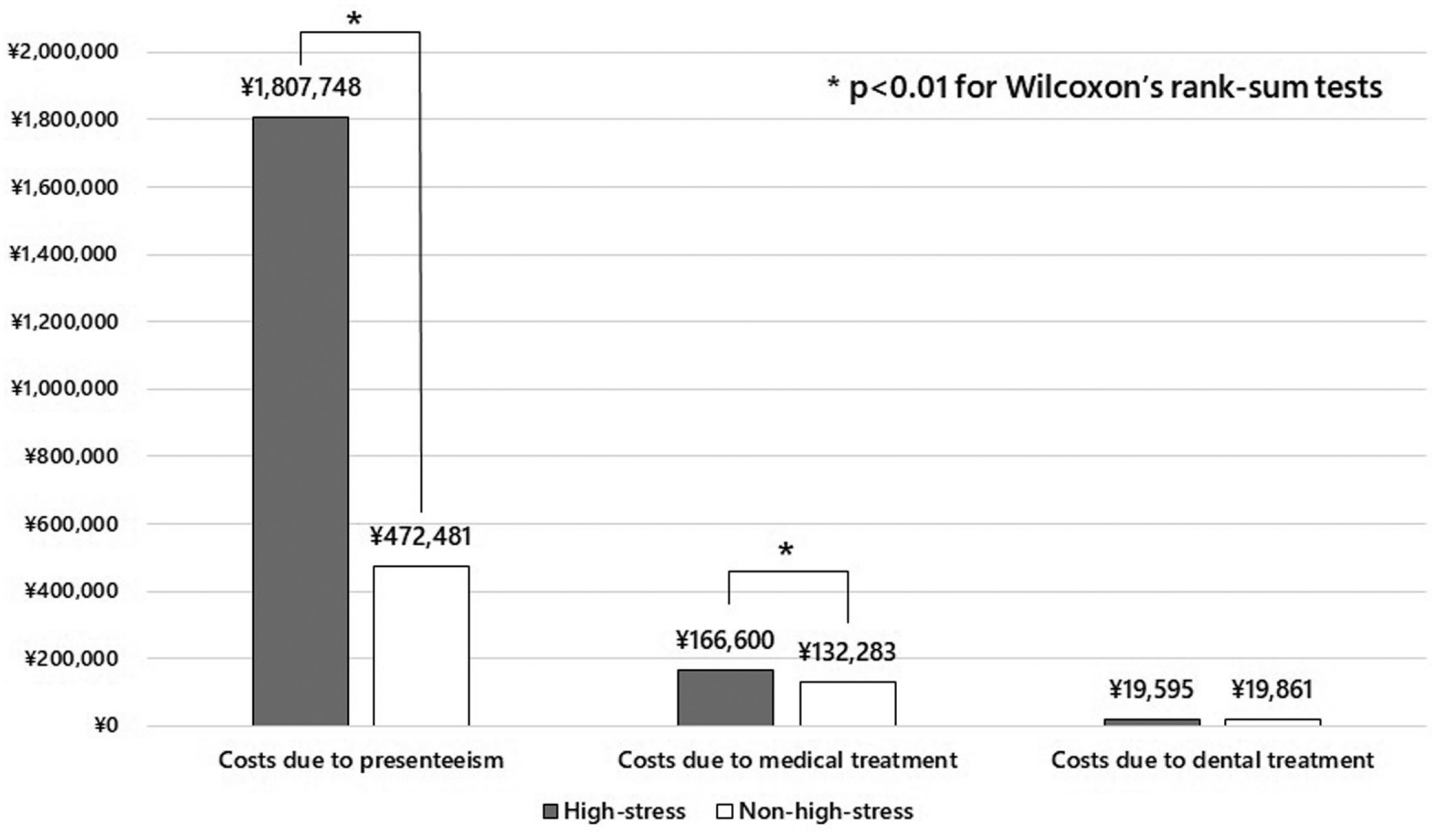
The costs due to presenteeism, medical treatment and dental treatment per person per year (Japanese Yen) stratified by high‐stress workers or non‐high‐stress workers according to the sum method.

As shown in Figure 2, using the sum method, the costs of presenteeism per person per year in the high‐stress and non‐high‐stress groups were JPY 1807748 and JPY 472481, respectively. The costs of medical treatment per person per year in the high‐stress and non‐high‐stress groups were JPY 166600 and JPY 132283, respectively. The costs of dental treatment per person per year in the high‐stress and non‐high‐stress groups were JPY 19595 and JPY 19861, respectively. The costs of presenteeism per year in the high‐stress group were higher than those in the non‐high‐stress group, *p* < .001, median JPY 902880 (inter‐quartile range [0–3 088 800]), median JPY 0 (inter‐quartile range [0–300 960]), respectively. The costs of medical treatment per year were also higher in the high‐stress than non‐high‐stress group, *p* = .013, median JPY 68150 (inter‐quartile range [20 580–177 080]), median JPY 52780 (inter‐quartile range [16 210–128 210]), respectively, while the costs of dental treatment per year were not significantly different between the high‐stress and non‐high‐stress groups, *p* = .858, median JPY 0 (inter‐quartile range [0–29 740]), median JPY 0 (inter‐quartile range [0–29 810]), respectively.

**FIGURE 3:**
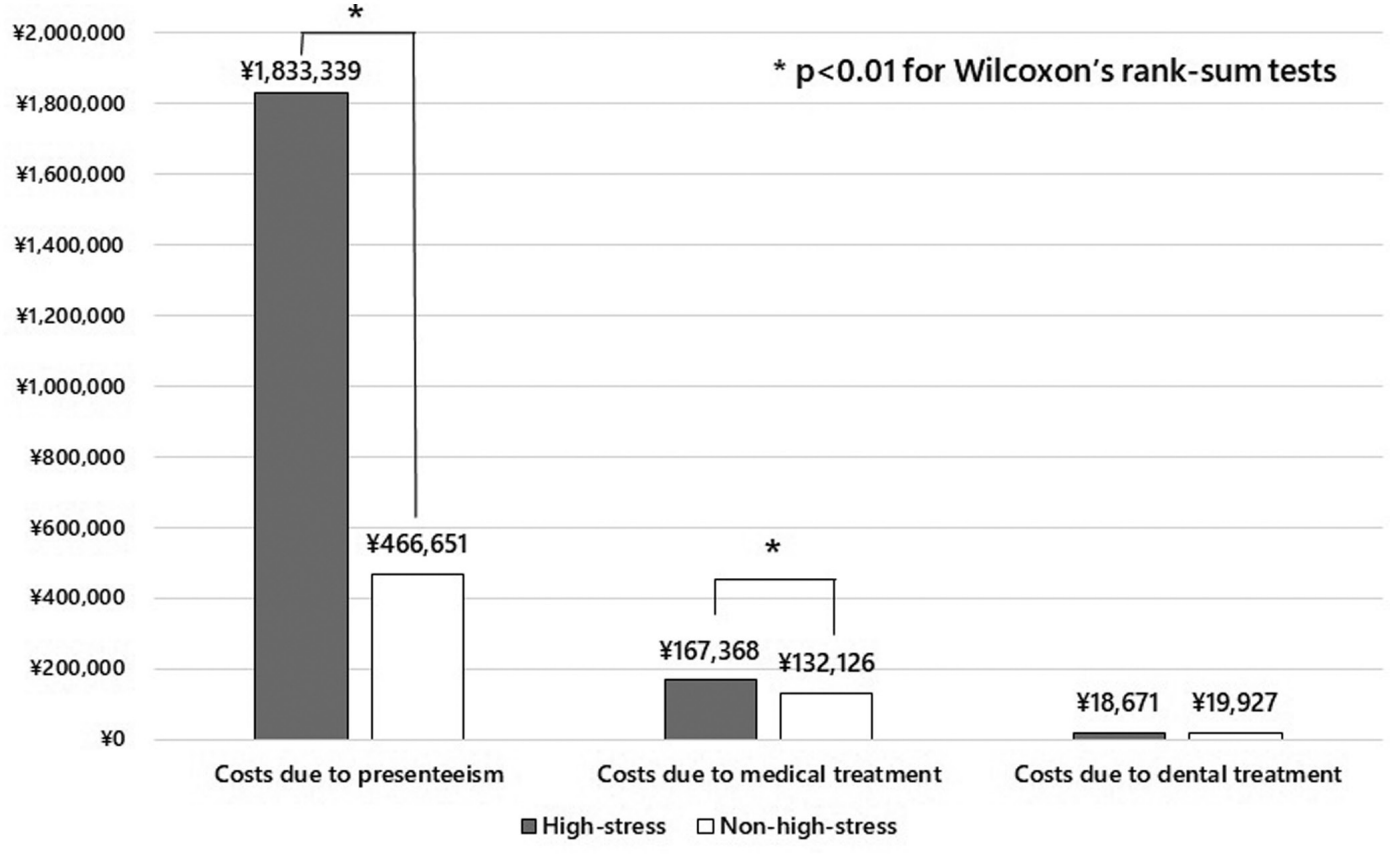
The costs due to presenteeism, medical treatment and dental treatment per person per year (Japanese Yen) stratified by high‐stress workers or non‐high‐stress workers according to the score converted method.

As shown in Figure 3, in the score converted method, the costs of presenteeism per person per year in the high‐stress and non‐high‐stress groups were JPY 1833339 and JPY 466651, respectively. The costs of medical treatment per person per year in the high‐stress and non‐high‐stress groups were JPY 167368 and JPY 132126, respectively. The costs of dental treatment per person per year in the high‐stress and non‐high‐stress groups were JPY 18671 and JPY 19927, respectively. The costs of presenteeism per year in the high‐stress group were significantly higher than those in the non‐high‐stress group, *p* < .001, median JPY 875952 (inter‐quartile range [0–3 193 344]), median JPY 0 (inter‐quartile range [0–300 960]), respectively. The costs of medical treatment per year were also significantly higher in the high‐stress group than those in the non‐high‐stress group, *p* = 0.007, median JPY 67375 (inter‐quartile range [23 870–169 520]), median JPY 52805 (inter‐quartile range [15 950–128 995]), respectively. The costs of dental treatment per year were not significantly different between the high‐stress and non‐high‐stress groups, *p* = .823, median JPY 0 (inter‐quartile range [0–28 830]), median JPY 0 (inter‐quartile range [0–29 905]), respectively.

## 4 DISCUSSION

The total costs due to presenteeism and medical and dental treatment per high‐stress worker were JPY 1994 thousand using the sum method and JPY 2019 thousand using the score converted method. These costs were JPY 1369 thousand and JPY 1401 thousand higher, respectively, than those associated with non‐high‐stress workers. In addition, presenteeism accounted for more than 90% of the total cost associated with high‐stress workers.

Previous studies have reported that occupational stress and mental health problems are major factors in presenteeism.^21^, ^22^ It has also been reported that approximately 75% of high‐stress workers classified using the stress check survey exhibited mild work functioning impairment as assessed by the Work Functioning Impairment Scale,^26^ and productivity loss due to presenteeism as assessed the work limitation questionnaire.^25^ In addition, it has been shown that presenteeism‐related losses account for a large proportion of losses caused by worker health problems, including presenteeism, sickness absence, and medical costs.^20^ Thus, the results of this study confirmed the findings of previous studies.

In Japan, stress check programs are implemented as a legal obligation for employers. The current results revealed a difference in losses of approximately 1400 thousand JPY between a worker classified as high‐stress using the stress check survey and a worker classified as non‐high‐stress. This result demonstrates how the results of the stress check surveys can have an impact on business performance, as measured using economic indicators. In the development of occupational health and safety and workplace health promotion, the leadership support of management and supervisors and the participation of workers are indispensable.^31^, ^32^ It is necessary for employees in these positions to show leadership in mental health measures in the workplace, and it is also necessary to make efforts to improve the workplace environment with workers' participation. The current findings may be helpful for explaining the necessity of implementing programs to improve the workplace environment as part of corporate management.

In this study, losses due to presenteeism were calculated in terms of economic value. The calculations were performed using the method of multiplying labor costs by the time of loss due to presenteeism. This method is called the human capital method.^33^ This method is often used in health economics, but it has been noted that it tends to overestimate losses because it does not take into account employee replaceability and other factors.^33^ In addition, the method by which we assessed presenteeism has proven to be poorly reliable and poorly related to actual work output.^34^ Therefore, the careful discussion is needed regarding the monetary equivalent of presenteeism. Hence, it is necessary to clearly show the formulas by which the monetary conversion was performed, and all formulas are shown in this study. We compared the results of our study with the previous study^25^ using the method and the formulas of cost calculation used in our study. Assuming an hourly wage of JPY3,300 and 247 working days used in our study, the average annual wage for fiscal year 2018 was calculated as follows: Annual labor costs = JPY 3300 × 8 (working hours per day) × 247 days = JPY 6520800. In the previous study,^25^ presenteeism was assessed using the work limitation questionnaire. The productivity loss for the high‐stress group was 9.72%, with a loss of 634 thousand JPY, while the productivity loss for the non‐high‐stress group was 4.92%, with a loss of 321 thousand JPY. The difference was 313 thousand JPY. Thus, it should be noted that the amounts differ depending on the method of valuation, but by stating the calculation method (formula) and the results together, comparisons can be made. We thought it was worth being comparable.

The method used in the current study can be applied in Japanese companies where the stress check system has been introduced. On the basis of the current findings, we recommend that presenteeism measurement items are added to the stress check survey. Thus, in addition to assessing the stress level and the percentage of high‐stress workers in the workplace, the amount of loss in productivity caused by presenteeism can also be expressed and continuously monitored in the stress check survey. However, even if no presenteeism measure is added, the current method can be used to calculate the amount of loss caused by presenteeism in each workplace. This measure is calculated by multiplying the number of high‐stress workers in each workplace by the average loss due to presenteeism in the present study. Regarding the method for assessing high‐stress workers, the stress check manual published by the MHLW provides two options: the sum method and the score converted method. The present study demonstrated the economic loss associated with high‐stress individuals, as assessed using the two methods, which can be applied in accord with the actual situation of each company. However, the current results are based on data from a specific company, and the average salary per hour per person needs to be adjusted to the actual situation of each company.

The current study involved several limitations that should be considered. First, we assessed presenteeism by the method used in the previous studies,^29^, ^30^ but the method has not been adequately validated with actual work performance. This is a common issue among several questionnaires that measure presenteeism.^34^ However, presenteeism refers to a decrease in productivity at work due to health problems,^35^ not a comprehensive work productivity. A previous study found an association between presenteeism as measured by this method and quality of life.^36^ We believe that it is highly necessary to verify the validity of the results with actual productivity in the future. Second, because this study was limited to only one company, the generalizability of the findings may be limited. The target company in this study was a large organization that works together with the health insurance society to promote workers' health. Thus, it is possible that the costs of medical and dental treatment and productivity losses due to presenteeism were underestimated compared with the general population. Third, the decline in productivity caused by health problems generally includes absenteeism in addition to presenteeism. However, in Japan, absenteeism caused by illness is often covered by paid leave, making it difficult to ascertain, so it was not included in the results of this study. In addition, long‐term absenteeism, which is often expressed as disability, was not included because human resource management data for the same period were not available. It has been previously reported that high stress in the stress check survey is a risk factor for long‐term absenteeism.^27^ Therefore, the present study may have underestimated the economic loss related to high‐stress workers. Fourth, because both the stress check and the measurement of presenteeism were based on self‐administered questionnaires, self‐selection bias and recall bias may have affected the current results.

However, despite these limitations, this study is the first to evaluate health‐related costs using data from stress check surveys administered in accordance with laws and regulations. As mentioned above, in the current situation, in which efforts to improve the work environment based on the results of stress check surveys are limited, we believe that the findings of this study are important for enhancing the awareness of employers and promoting the effective use of the stress check program.

## 5 CONCLUSIONS

The costs associated with presenteeism and medical treatment among high‐stress workers were higher than the costs among non‐high‐stress workers, whereas the costs associated with dental treatment were not. To motivate employers to improve stressful work environments, it is recommended that presenteeism measurement items are added to the stress check survey, and that the methods used in this study are used to calculate the loss associated with high‐stress workers in Japanese companies. However, we must be careful in interpreting absolute presenteeism loss amounts because they are poorly reliable and valid.

## AUTHORS CONTRIBUTIONS

Tomohisa Nagata and Koji Mori conceived and coordinated the project; Tomohisa Nagata, Ryotaro Ito, Kenji Fujimoto, and Koji Mori analyzed the data and prepared the first draft; Masako Nagata, Kiminori Odagami, Shigeyuki Kajiki, and Shinya Matsuda revised the manuscript; all authors commented on the draft of the report.

## DISCLOSURE

Ethical approval: This study was approved by the Ethics Committee of the University of Occupational and Environmental Health, Japan (reference No. H26‐026). Informed consent: Informed consent was obtained via an online form. Registry and the registration no. of the study/trial: N/A. Animal studies: N/A. Conflicts of interest: The authors declare no conflicts of interest associated with this manuscript.

## Data Availability

The data that support the findings of this study are available from the corresponding author, Tomohisa Nagata, upon reasonable request.
